# Cell migration in the developing rodent olfactory system

**DOI:** 10.1007/s00018-016-2172-7

**Published:** 2016-03-18

**Authors:** Dhananjay Huilgol, Shubha Tole

**Affiliations:** 1 Department of Biological Sciences, Tata Institute of Fundamental Research, Mumbai, India; 2 Present Address: Cold Spring Harbor Laboratory, Cold Spring Harbor, USA

**Keywords:** Olfactory, Vomeronasal, Migration, Domains, Evolution, Neocortex

## Abstract

The components of the nervous system are assembled in development by the process of cell migration. Although the principles of cell migration are conserved throughout the brain, different subsystems may predominantly utilize specific migratory mechanisms, or may display unusual features during migration. Examining these subsystems offers not only the potential for insights into the development of the system, but may also help in understanding disorders arising from aberrant cell migration. The olfactory system is an ancient sensory circuit that is essential for the survival and reproduction of a species. The organization of this circuit displays many evolutionarily conserved features in vertebrates, including molecular mechanisms and complex migratory pathways. In this review, we describe the elaborate migrations that populate each component of the olfactory system in rodents and compare them with those described in the well-studied neocortex. Understanding how the components of the olfactory system are assembled will not only shed light on the etiology of olfactory and sexual disorders, but will also offer insights into how conserved migratory mechanisms may have shaped the evolution of the brain.

## Introduction: cell migration in the developing forebrain

In the developing nervous system, neurons are born at specialized sites where progenitors reside. The central nervous system arises from the neural tube, and proliferating progenitors line the ventricle forming the ventricular zone (VZ). Neurons and glia, the postmitotic progeny of these cells, must often migrate to distant destinations to form mature brain structures. Cell migration is critical for proper circuit formation and functioning of the brain. Aberrant neuronal migration has been implicated in disorders such as epilepsy [1, 2], schizophrenia [3, 4], autism [5, 6] and in severe learning disabilities [7, 8]. Studying cell migration is therefore imperative for our understanding of brain development and the etiology of neurodevelopmental disorders.

The principles of neuronal migration are largely similar throughout the brain to the extent that they are currently understood. That said, neurons of the telencephalon and diencephalon, which together form the cerebral hemispheres, display extremely complex trajectories and elaborate migratory movements to reach their final destinations. As a result of these migrations, the telencephalon produces an array of diverse structures subserving distinct functions: the olfactory bulbs (OBs), the cerebral cortex, the basal ganglia, and the amygdaloid complex. The diencephalon forms the thalamus and the hypothalamus.

**Fig. 1:**
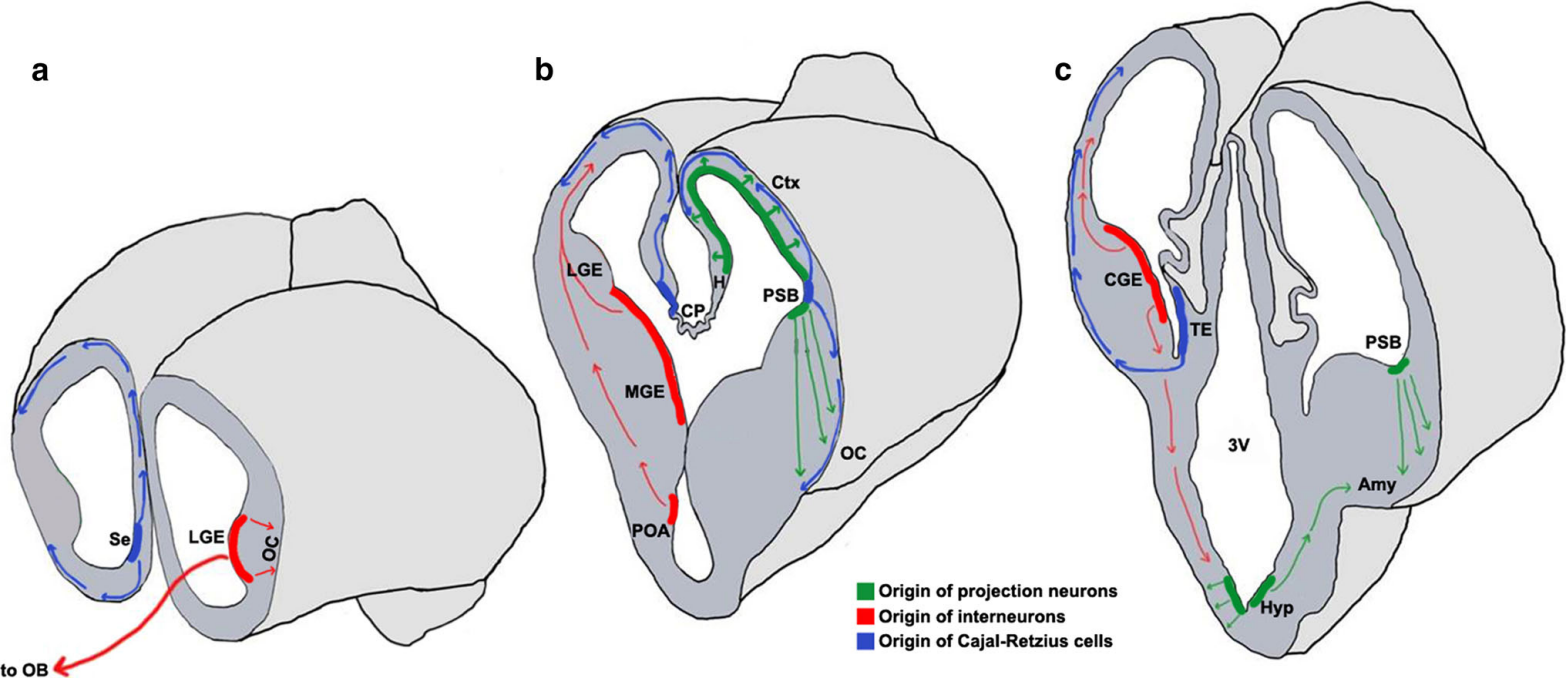
Neuronal migrations in the embryonic forebrain [13, 22, 23, 25–27, 29, 36, 67, 303–305]. Schematics representing the mouse brain at embryonic day (E)12.5 sectioned in the coronal plane at the rostral (**a**), mid (**b**), and caudal (**c**) levels. Domains of origin and migration routes for projection neurons (*green*), interneurons (*red*), and Cajal–Retzius cells (*blue*) are illustrated. *Colored bands* represent the ventricular zone and progenitors residing therein; *arrows* represent the route and direction of migration. *3V* third ventricle, *Amy* amygdala, *CGE* caudal ganglionic eminence, *CP* choroid plexus, *Ctx* cortex, *H* hippocampus, *Hyp* hypothalamus, *LGE* lateral ganglionic eminence, *MGE* medial ganglionic eminence, *OB* olfactory bulb, *OC* olfactory cortex, *POA* pre-optic area, *PSB* pallial–subpallial boundary, *Se* septum, *TE* thalamic eminence

Cell migration in the brain may be broadly categorized based on the orientation of the migration trajectory with respect to the ventricular surface [9, 10]. Neurons may migrate either radially outward from this surface, or tangentially, in a direction orthogonal to the radial axis. Projection neurons, which are typically excitatory, primarily exhibit radial migration (Fig. 1) [10–12]. Interneurons, which are typically inhibitory, undertake tangential migration for much of their journey (Fig. 1) [9, 13–15].

These two broad categories of migration are regulated by a spectrum of complex mechanisms that are well worth understanding, since it is cell migration that literally builds and shapes brain structures. Here, we review the migrations that contribute to the different components of the olfactory system in rodents. We compare and contrast the mechanisms underlying these migrations with those utilized in the well-studied neocortex and highlight features unique to the olfactory system. We conclude with developmental, disease, and evolutionary perspectives on cell migration in this system.

## The main and accessory olfactory systems

The sense of smell is essential for a variety of behaviors such as mating, feeding, fear, and aggression. In rodents, the olfactory system has two distinct components: the main olfactory system, which is responsible for the sense of smell, and the vomeronasal system (VNS; also called the accessory olfactory system), which is essential for pheromone-based communication [16, 17]. These systems are tuned to discriminate between a variety of distinct odors and can do so at very low concentrations [18, 19]. Such efficient information processing requires the precise arrangement of a highly ordered circuit. In the sections below, we will examine the main and the accessory olfactory systems in terms of the cell migrations that create the mature circuits.

The olfactory system is unique among the sensory systems in how information enters the cortex. Whereas visual, auditory, and somatosensory input reaches the respective primary cortical areas via the thalamus, the olfactory cortex (OC) gets inputs directly via the OB. The OB is therefore the primary integration center of olfactory input in the brain.

### Domains of origin

Throughout the central nervous system, neuronal cell fate is specified based on the domain of origin of the postmitotic cells in the VZ. In the telencephalon, the dorsal (pallial) VZ produces excitatory neurons from molecularly distinct domains called the medial, dorsal, lateral, and ventral pallia (MP, DP, LP, and VP, respectively) [20]. The ventral (subpallial) telencephalon is divided into the lateral, medial, and caudal ganglionic eminences (LGE, MGE, CGE, respectively) and the VZ of these domains produces distinct categories of interneurons based on an intricate transcription factor-based code [21–25]. At the rostral end of the telencephalon, the VZ of the septum has pallial as well as subpallial domains [26, 27]. Just dorsal to the septum is the rostromedial telencephalic wall (RMTW), which, together with the neuroepithelium of the septum, constitutes a rostral source of neurons for the forebrain [28, 29].

Broadly, excitatory projection neurons are pallial, and inhibitory interneurons are subpallial in origin. The DP produces excitatory neurons of the neocortical sensory areas (visual, auditory, somatosensory), the motor cortex, and higher cortical areas. In contrast, the OC, which processes the sense of smell, is populated by excitatory neurons from the LP and VP [26, 30–32]. The boundary between the pallium and subpallium, called the pallial–subpallial boundary (PSB), gives rise to the lateral cortical stream (LCS), which contributes both excitatory and inhibitory neurons to the OC [28, 32–35]. Neurons in the LCS migrate along a radial glial palisade that extends from the VZ of the PSB to the pial surface [35, 36]. This migration has similarities with mechanisms known to operate in neocortical projection neurons. Migrating LCS cells require doublecortin (Dcx), Lis1 [37], and filamin A [38] to maintain a bipolar morphology. Electroporation of shRNA in rat embryos to knockdown *Dcx* or *Lis1* in the LCS leads to the aberrant accumulation of cells [37], similar to the effects of *Dcx* knockdown in the rat neocortex [39]. The LCS is not a unitary migration, however. It contains cells arising from multiple domains that lie on either side of the PSB, namely the LP, VP, and dorsal lateral ganglionic eminence (dLGE). A complex molecular code distinguishes the contributions of each domain of origin: cells arising in the LP express Tbr1, Emx1, and Pax6; the VP, Tbr1 and Pax6; and the dLGE, Pax6 and Dlx2 [26, 33, 34]. The persistent expression of Pax6 is a feature of migrating LCS cells that is not seen in the neocortex, wherein cells express Pax6 only when they are proliferating and turn off expression as they become postmitotic and commence migration [40–42].

The VZ of the LGE, MGE, and CGE contains discrete domains that generate inhibitory neurons that populate the entire telencephalon and also some diencephalic structures [23].

The domains of origin described above reside within the telencephalon. Other important sources of olfactory system neurons lie within the diencephalon or at structures located at the diencephalic–telencephalic boundary (DTB). These domains typically contribute to amygdaloid and hypothalamic nuclei that process olfactory information and will be discussed in later sections.

## The main olfactory system

**Fig. 2:**
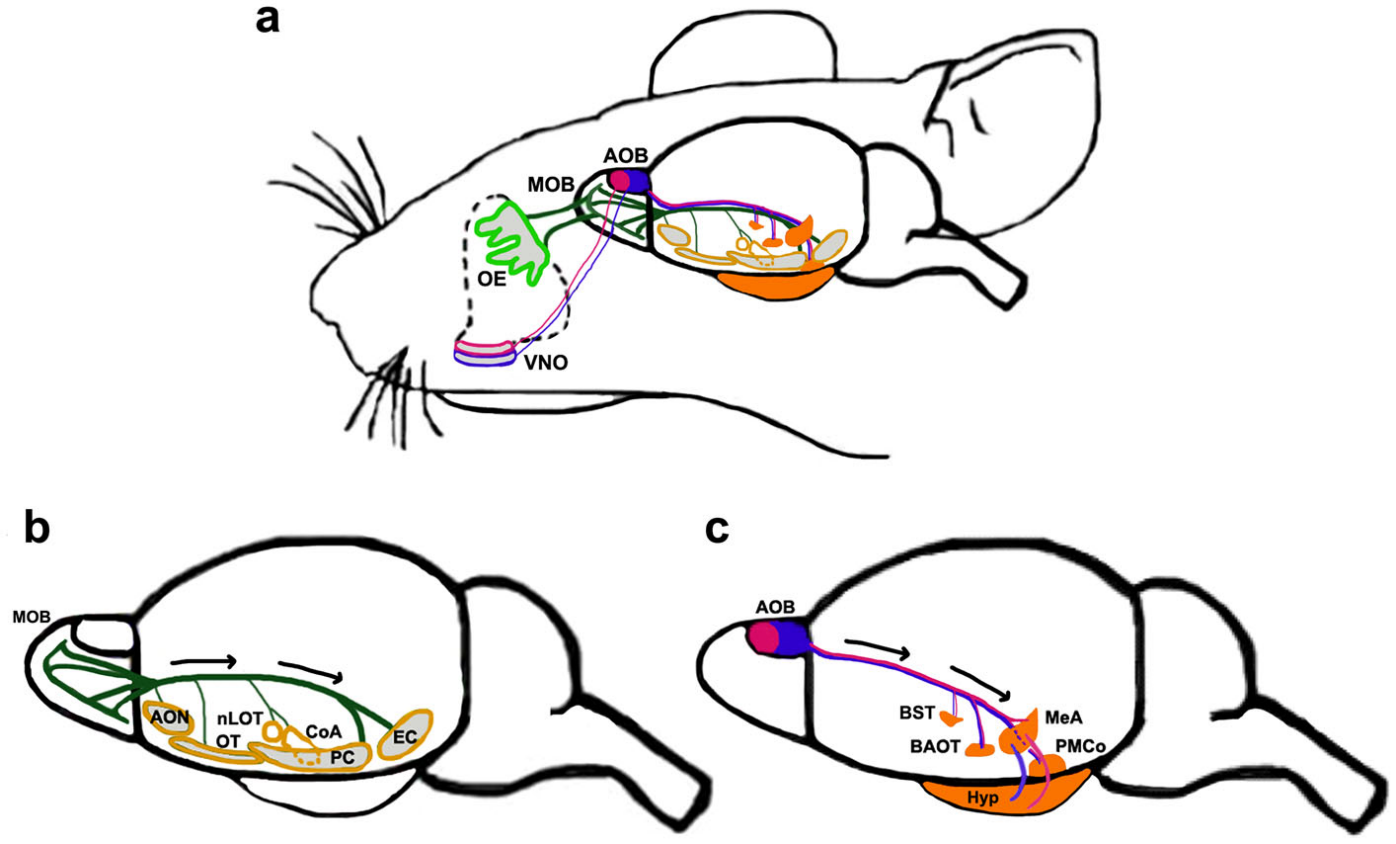
The main and accessory olfactory system [16, 43–46, 151, 189–192, 194, 205]. **a** Schematic depicting both systems of the mouse olfactory circuit. OSNs located in the OE (*light green*) project to the MOB, whereas VSNs in the VNO project to the AOB. Apical (*pink outline*) and basal (*blue outline*) VNO neurons project to the aAOB (*solid pink*) and pAOB (*solid blue*), respectively. **b** The main olfactory system. MOB M/T cells send their axons along the LOT in the direction of the *black arrows* to multiple targets (*yellow outlined structures*). These include different components of the olfactory cortex: the AON, OT, PC, EC, and the olfactory amygdaloid nuclei, CoA and nLOT. **c** The accessory olfactory system. Both the aAOB (*solid pink*) and the pAOB (*solid blue*) projection neurons send axons along the LOT (in the direction of the *black arrows*) to different parts of the vomeronasal amygdala, including the MeA, PMCo, and components of the extended amygdala, BST, BAOT (*solid orange structures*). *AOB* accessory olfactory bulb, *aAOB* anterior AOB, *pAOB* posterior AOB, *AON* anterior olfactory nucleus, *BAOT* bed nucleus of accessory olfactory tract, *BST* bed nucleus of stria terminalis, *CoA* cortical amygdaloid nucleus, *EC* entorhinal cortex, *Hyp* hypothalamus, *LOT* lateral olfactory tract, *MeA* medial amygdaloid nucleus, *MOB* main olfactory bulb, *nLOT* nucleus of the lateral olfactory tract, *OE* olfactory epithelium, *OT* olfactory tubercle, *PC* piriform cortex, *PMCo* posteromedial cortical nucleus, *VNO* vomeronasal organ

The main olfactory bulb (MOB) receives sensory input from olfactory sensory neurons (OSNs) in the olfactory epithelium (OE) via the olfactory nerve. Mitral/tufted (M/T) cells, the projection neurons of the MOB, receive OSN synapses and in turn project via the lateral olfactory tract (LOT) to the multiple components of the OC. The OC comprises five different regions, namely, the anterior olfactory nucleus (AON), the olfactory tubercle (OT), olfactory amygdala, piriform cortex (PC), and entorhinal cortex (Fig. 2) [43–46].

### The main olfactory bulb

MOB morphogenesis is carried out in two steps. The projection neurons are born first, in the VZ at the rostral tip of the telencephalon, from where they migrate outward to create a small protrusion, the MOB anlage, by embryonic day (E)12–13 in the mouse. This is closely followed by the entry of interneurons (granule cells and periglomerular cells) which begins from E14 [47–49]. Cells of the MOB display a laminar arrangement such that the M/T projection neurons, granule cells and periglomerular cells all occupy distinct layers. A defect in the development and organization of any of these populations can lead to a lack of MOB protrusion [50] and severe functional consequences that we discuss further in the “Disease perspectives” section of this review.

#### MOB projection neurons

The M/T cells share interesting features with neocortical projection neurons. They are derived from pallial (dorsal telencephalic) progenitors at the local MOB VZ [51], arising from Pax6-positive radial glia [52], and their migration follows an inside-out pattern. Although distinct laminae that are characteristic of the neocortex have not been described within the M/T layer of the MOB, neurons residing deepest in this layer are born first, and later born neurons migrate past these and settle at more superficial locations [48, 50].

**Fig. 3:**
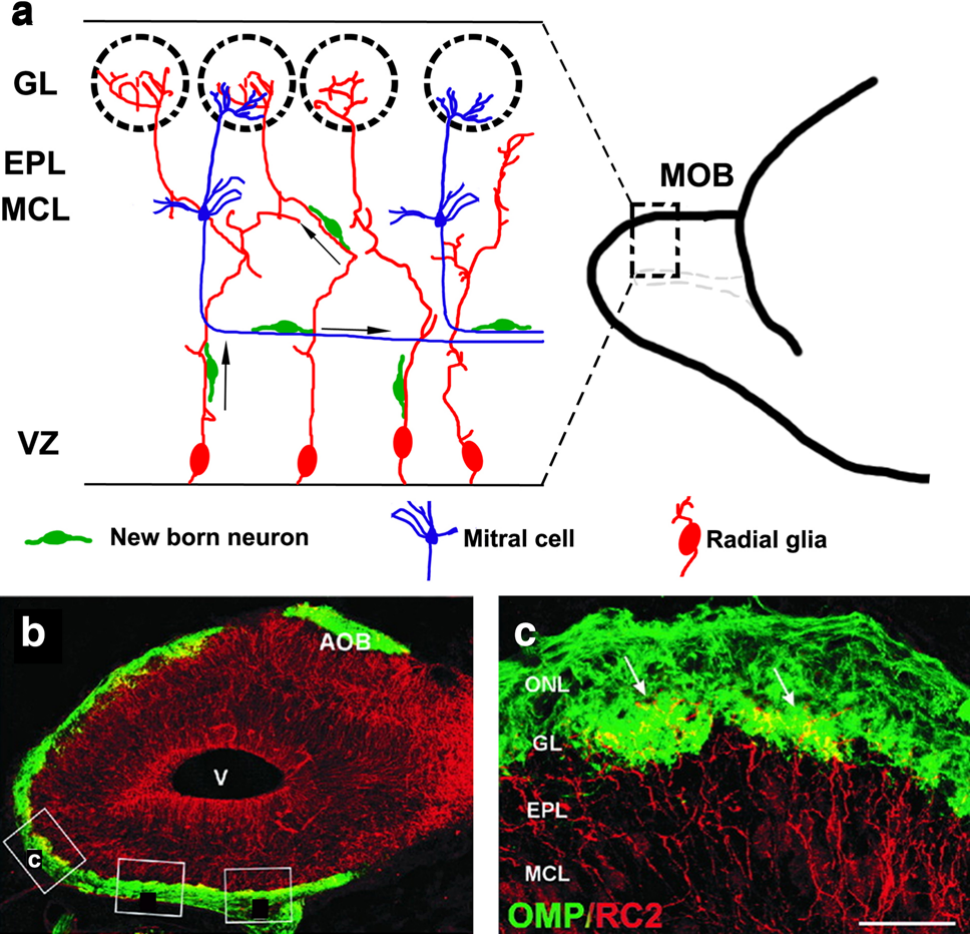
Migration of new neurons within the MOB [51, 52, 58]. **a** Schematic showing a sagittal section of an embryonic day (E)18.5 MOB. The radial glia (*red*) are convoluted and intertwined, with their endfeet merging in the glomeruli or in the EPL. The cell bodies of M/T cells (*blue*) are in the MCL, and their axons extend parallel to the ventricular zone. Newborn neurons (*green*) migrate radially to their destined laminar positions using the radial glia, or tangentially using M/T cells axons as scaffolds. **b** Confocal image of an E18.5 mouse MOB sagittal section immunostained for a radial glial marker, RC2 (*red*), and an OSN marker, the olfactory marker protein (OMP, *green*). **c** Magnified view of *boxed area* in **b** showing radial glial endfeet (*arrows*) penetrating glomeruli formed by OSN axons. *Scale bar* in **c** is 400 μm. *Additional boxes* in **b** are from the original artwork in [58]. *AOB* accessory olfactory bulb, *EPL* external plexiform layer, *GL* glomerular layer, *MCL* mitral cell layer, *MOB* main olfactory bulb, *ONL* olfactory nerve layer, *OSN* olfactory sensory neuron, *V* ventricle, *VZ* ventricular zone. The images in **b**, **c** are from Fig. 1 of [58], copyright 2001 Wiley-Liss, Inc. Reprinted with permission

Transcription factors important for neocortical development, *Pax6* and *Lhx2*, are required to regulate M/T cell migration in the OB. Loss of either of these results in no OB, but instead a misplaced, lateral olfactory bulb-like structure (OBLS) [53–56] that includes MOB and accessory OB (AOB) components. In addition to these shared genetic mechanisms required for the development of both the MOB and the neocortex, MOB-specific mechanisms also exist: transcription factors *AP2*-*epsilon* [55], *Arx* [56], and *FezF1* [57] are necessary for proper orientation of M/T cells and organization of MCL, but are not known to be required for the development of neocortical projection neurons. Finally, there are some intriguing differences in the cellular and molecular mechanisms that mediate neocortical and M/T cell migration. Unlike in the neocortex, where radial glia have a uniform parallel arrangement, MOB radial glia display complex branched and intertwined morphologies, the function of which is not entirely clear (Fig. 3) [58]. Furthermore, newborn M/T neurons exhibit radial as well as tangential migration. In particular, later born cohorts migrate tangentially using the axons of earlier born cells to reach their proper location in the MCL (Fig. 3) [51]. Additionally, postmitotic M/T neurons express both Tbr2 and Tbr1 [52], unlike neocortical neurons, which switch off Tbr2 upon becoming postmitotic and express only Tbr1 [42]. Tbr2 expression in postmitotic neurons in the MOB is necessary for the proper migration of M/T cells and their organization in the MCL [59].

#### MOB interneurons

MOB interneurons, like cortical interneurons, are born in the subpallium and undertake a tangential migration route to reach their destination [22, 60, 61]. Whereas cortical interneurons arise from the MGE and CGE [21–25], MOB interneurons arise from the dorsal segment of the lateral ganglionic eminence (dLGE), the LGE [62], and the septum [63]. Interneuron precursors born in these domains commence a rostral, tangential migration into the MOB. One portion of this migration, from the dLGE, continues into adulthood, constituting what is known as the rostral migratory stream (RMS) [64].

There is a large diversity of MOB interneuron subtypes, in terms of morphology and neurochemical content [65, 66] as is the case for cortical interneurons [67, 68]. There is also a surprising temporal and spatial control of MOB interneuron diversity, which includes up to seven distinct subtypes based on neurochemical and neuropeptide markers [65]. Different interneuron subtypes are generated depending on the age of the animal, with particular subtypes being born at specific embryonic or postnatal stages [65, 69]. Spatially distinct progenitors with unique molecular signatures produce the diversity of MOB interneurons. The LGE produces interneurons from *Gsh2*-positive progenitors which are also *Pax6* positive (from the dLGE) [32], or *Dlx2* positive (from the rest of the LGE) [70]. Conditional removal of *Pax6* alters the postnatal production of dLGE-derived interneurons [71]. There is also a locally generated pool of *Pax6* expressing progenitors in the OB VZ, which produces both GABAergic granule cells and dopaminergic periglomerular interneurons [70, 72]. An unusual pool of MOB interneurons arises from the pallial *Emx1* lineage. These progenitors arise from E15 and integrate with the *Dlx2* expressing LGE progenitors within the striatal germinal zone. In this new subpallial location, these cells begin to express *Dlx2* and then contribute to the MOB interneuron pool through adulthood [72, 73]. The MOB therefore displays an unexpected complexity and temporal dynamics in the molecular identity of its interneuron population.

Embryonic MOB interneurons utilize similar molecular mechanisms to those employed by cortical interneurons to regulate their migration. These include transcription factors Dlx1, Dlx2, and Mash1 and also the Robo–Slit and neuregulin signaling systems [74–76].

From postnatal to adult stages, the progenitors of the RMS migration reside in the anterior subventricular zone (aSVZ) which is derived from the embryonic dLGE [22]. Despite this developmental continuity of the domain of origin, distinct mechanisms are utilized by embryonic versus adult cells for migration. In the postnatal and mature RMS, interneuron precursors, or neuroblasts, migrate in a closely associated neurophilic or chain migration pattern, along blood vessels with the aid of astrocytes which form a glial tunnel ensheathing the migrating cells [77–82]. These astrocytes are detected only by early postnatal ages and are not seen embryonically [83]. The polysialylated form of neural cell adhesion molecule (PSA-NCAM) is necessary for chain migration and is therefore expressed robustly by the neuroblasts from perinatal stages [84–86]. In addition, adhesion molecules such as integrins are differentially expressed during the migration of embryonic versus adult neuroblasts; α1 and β1 subunits are expressed in the embryo, whereas αv, β3 and β6 subunits are expressed in the adult [87, 88]. An unusual mechanism is employed in the last phase of migration when the cells reach the MOB and must migrate radially outward into their destined layers to differentiate into mature interneurons [89]. For this stage of migration, adult neuroblasts are guided along blood vessels, in contrast to embryonic cells which use radial glial-guided migration [90].

OB projection neurons, interneurons/neuroblasts therefore demonstrate the use of cellular substrates other than radial glia for their migration, i.e., the axons of M/T neurons, other neuroblasts in chain migration, and blood vessels. This contrasts with the neocortex in which radial glia are the only reported cellular substrates utilized by projection neurons and interneurons [9, 91–94]. About 50 % of MGE-derived interneurons can utilize axons for their migration in vitro [95, 96], but there is no direct evidence of axon-mediated migration of cortical interneurons in vivo. Another major point of difference with cortical interneurons is that from perinatal stages, RMS migration comprises neuroblasts-specified precursors that will produce interneurons, but which retain proliferative capability and indeed do proliferate during their chain migration. This unusual feature is seen in only a few sites in the entire central nervous system—dentate granule cells, cells from the olfactory placode (OP), and cells migrating along the LCS are the only other populations that exhibit simultaneous proliferation and migration of neuroblasts [35, 97–100]. The latter two populations are part of the olfactory migrations described in this review.

#### Olfactory placode: a cell source for the OB

The OE, a derivative of the OP in the snout, is one of the few regions outside the neural tube that generates neurons. The OE produces OSNs which relay sensory information to the OB throughout the life of an organism [101–105]. The OP also produces multiple cell types that populate the OB. These cells, termed the “migratory mass” (MM) [106], migrate together with the OSN axons as they extend from the OE toward the OB bundled in the olfactory nerve [106–109]. The MM is well characterized in rodents and chick, and known to contain cells with molecularly distinct identities. Differentiated cells within the mesenchyme of mouse embryos are observed as early as E10–10.5 [110]. Cells of the MM express different combinations of markers such as Doublecortin, *Notch1* and its effector *Hes5*, Delta/Notch-like EGFR receptor (DNER), OMP, Lhx2, and GnRH [110, 111]. The MM includes putative guidepost neurons for OSNs; olfactory ensheathing cells (OECs) and their precursors; neurons expressing the olfactory marker protein (OMP); and several other distinct cell types expressing Dlx5, Six1, NCAM, GAP43, or vGlut2 whose fate and function are not well understood [100, 106, 110, 112–123].

The OECs ensheath the OSN axons throughout their growth, during their extension through the cribriform plate, and into the olfactory nerve layer and glomerular layer of the OB [100, 124–126]. OECs express BLBP and S100β, and have a range of functions in the development and immunity of the olfactory system. OECs envelope the OSN axons along their entire length forming a complex extracellular matrix containing laminin and fibronectin, express cell adhesion molecules such as PSA-NCAM and N-cadherin, produce neurotrophic factors including the p75 neurotrophin receptor and nerve growth factor, and express guidance cues such as ephrin B2 and semaphorin 3A. All these molecules promote axon growth and fasciculation [100, 127–135]. OECs also release soluble factors such as fibroblast growth factor, FGF2, which are thought to regulate the proliferation and differentiation of OSN progenitors [136–138]. They also participate in innate immunity and thereby protect the peripheral olfactory system from pathogens. They release neuropeptide Y, show inflammatory signaling cascades in response to bacterial trigger, and can lyse bacteria following endocytosis [139–144]. A subpopulation of OECs are precursors that express Sox2 and nestin, and continue to proliferate during migration.

An intriguing guidance role for OECs is suggested by reports of OR expression in this population. Each OSN axon expresses a specific individual odorant receptor (OR), which serves not only as a receptor for odorant molecules, but also guides the OSN axon to a specific glomerulus within the OB where it synapses with an M/T cell [145–148]. Interestingly, OMP expressing cells among the OECs contain a subpopulation that also expresses individual ORs. A curious feature of these cells is that they associate with OSN fibers that express the same OR. This suggests the speculative possibility that these cells may be involved in the regulation of OSN guidance via as yet unidentified mechanisms that may include a guidepost-like role [120, 149]. Though OMP expression is not found in birds, OECs that express individual ORs and associate with the growing OSN fibers have been reported in chick [118, 119], suggesting that this population may be conserved in evolution.

OECs may have additional roles into adulthood. OECs can attract RMS interneuron progenitors over short length scales in vitro, comparable to the distance interneurons travel during their radial migration phase after they enter the OB. It is possible that OECs may provide such an activity in vivo as well [100, 150].

A special class of differentiated neurons within the MM is the gonadotropin-releasing hormone (GnRH) neurons, which migrate past the OB to the hypothalamus [108, 109]. These GnRH neurons will be discussed further in “Hypothalamic nuclei”.

In summary, the MM is an interesting mix of dividing, postmitotic, and fully differentiated cells. The identities and functional implications of the diversity within this population are yet to be completely understood, but it reveals the broad range of regulatory and functional contributions of placode-derived neurons and non-neuronal cells to the development of the olfactory system.

#### Migration of “lot cells”

**Fig. 4:**
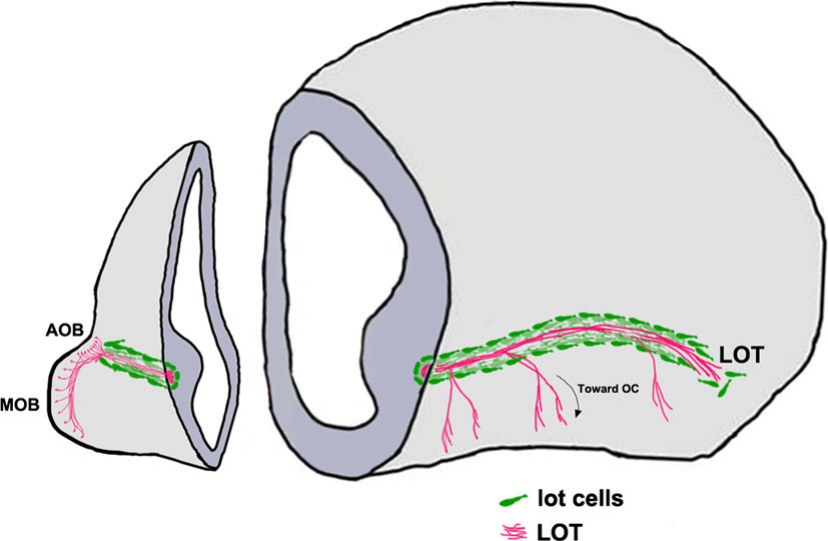
“Lot cell” array and formation of the LOT [152, 153]. Schematic representing one hemisphere of an embryonic day (E)14.5 mouse brain. The projection neurons of the MOB and the AOB extend their axons along the LOT and innervate different olfactory cortical and vomeronasal structures. The “lot cells” (*green*) form a “permissive corridor” along the lateral face of the telencephalon through which the LOT axons (*pink*) grow. *AOB* accessory olfactory bulb, *LOT* lateral olfactory tract, *MOB* main olfactory bulb

Axons from the MOB and AOB project to their targets via the LOT [151]. These are restricted to a tight corridor created by a group of guidepost neurons known as the “lot cells” (Fig. 4) [152]. The lot cells are an intriguing population, sharing molecular features with Cajal–Retzius cells and posterior AOB (pAOB) M/T cells, yet serving a highly specialized function in olfactory development. Lot cells are believed to arise in the dorsal neocortical VZ; from here, they migrate ventrally and tangentially along the telencephalic surface and finally align themselves at the PSB along the entire rostrocaudal extent [153]. A recent study (Ruiz-Reig et al., under revision, cerebral cortex) offers evidence that lot cells may arise from a different source, the thalamic eminence (TE). This transient structure located at the DTB is the source of several distinct migratory populations described later in this review. Intriguingly, a subpopulation of the TE-derived lot cells may later differentiate into pAOBM/T cells (Ruiz-Reig et al., under revision, cerebral cortex), which highlights a new evolutionary interpretation of the pAOB, discussed at the end of this review. Proneural transcription factors neurogenin 1 and 2, necessary for patterning and cortical neuron specification [154, 155], are required for the differentiation of lot cells [156].

The lot cells assemble at the prospective LOT position prior to the incoming OB M/T cell axons forming the LOT. Proper alignment of the lot cells is essential for guiding the LOT. Ablating these cells using 6-hydroxydopamine disrupts the formation of a proper LOT [152].

It is therefore not surprising that complex cell-autonomous and cell non-autonomous mechanisms guide the positioning of the lot cell array. Netrin1/DCC guidance is necessary for lot cell alignment at the PSB [157]. Semaphorin 3F, which is expressed in the mantle of the lateral telencephalon, restricts the lot cells to the telencephalic surface [158]. The lot cell array is profoundly disrupted when transcription factors Gli3 or Lhx2 are lost [54, 153, 157]. These transcription factors may directly control lot cell migration via cell-autonomous mechanisms, or indirectly via regulation of signaling at the PSB. Transcription factor Gli3 is required for dorsoventral patterning, such that the subpallial component of the PSB expands dorsally in the *Gli3* mutant [159, 160]. Disruption of lot cell array in this mutant is cell non-autonomous [153, 157] and may be due to perturbed signaling cues at the PSB. Loss of transcription factor Lhx2 does not affect the position of the PSB [161], but causes upregulation of *Semaphorin 6A* expression in the lateral telencephalic region where the lot cells accumulate, which may underlie the profoundly disrupted lot cell array in this mutant [54].

### Olfactory cortex: many structures and multiple migrations

The OC extends along the entire rostrocaudal length of the ventral telencephalon and consists of five structures—the AON, OT, PC, entorhinal cortex, and the olfactory amygdala [44]. OB projection neurons make connections with different rostrocaudal portions of the OC depending on their location in the OB and birth order. Mitral cells residing in the ventral OB project to the OT, whereas those residing in the dorsal OB preferentially project to the PC [162, 163]. The birth order of these neurons plays a role in determining the strength of projections to particular OC areas, such that later born cells project more axons to the OT than those born earlier [51]. An additional complexity is that within the class of projection neurons, mitral cells connect with more posterior and tufted cells with more anterior OC regions [163, 164].

The components of the OC display either a nuclear or a trilaminar cortical organization. Of the latter type, the trilaminar PC is best studied in terms of its cytoarchitectonics and connectivity. Layer 1 is a cell-sparse zone and contains dendrites from the underlying cellular layer 2, long distance axons from the OB bundled in the LOT, and intracortical feedback connections. Layer 2, the principal cellular layer, is densely packed with pyramidal neurons and granule cells. Layer 3 has sparse pyramidal and polymorphic cells with no apical dendrites. It is primarily involved in intracortical communication rostrocaudally within the OC [165].

The olfactory amygdala, which receives input from the MOB comprises two amygdaloid nuclei: the cortical amygdala (CoA) and nucleus of the lateral olfactory tract (nLOT) [166]. Both these nuclei are considered to be “cortical” since they appear laminated and have radially oriented pyramidal neurons [167].

OC receives cells from multiple regions of the forebrain, some of which originate at E10.5 [28, 29], the same time as the Cajal–Retzius cells and subplate cells which are the earliest born cells of the neocortex. OC neurogenesis in the rodent embryo continues until late gestation [168]. The deeper neurons of OC (layer 3) are born earlier than the superficial neurons (layer 2), particularly in the PC [168–170]. Therefore, the OC displays a rudimentary inside-out pattern of neurogenesis, similar to the neocortex [171].

#### Migrations to the components of the OC

**Fig. 5:**
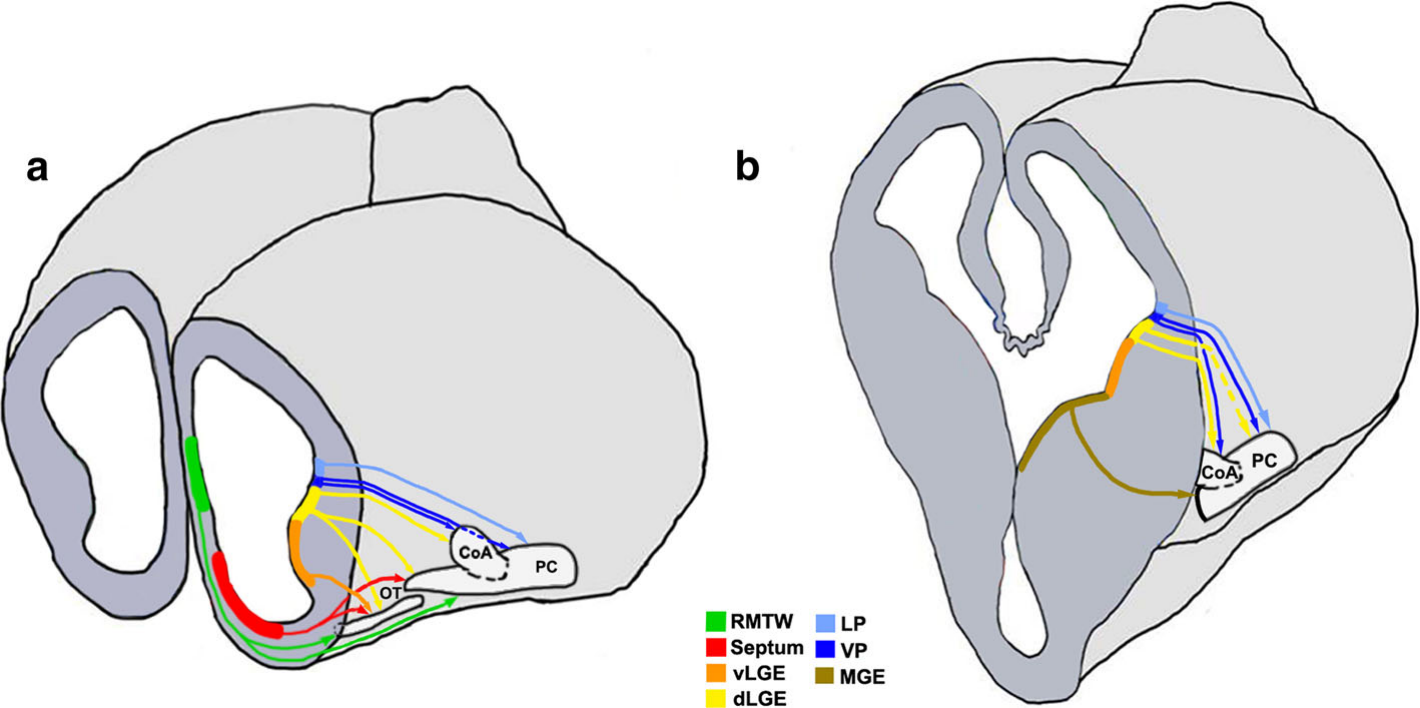
Cell migrations to the olfactory cortex [26, 29, 30, 34, 35, 37, 172]. Schematics representing an embryonic day (E)12.5 brain sectioned at rostral (**a**) and mid (**b**) levels in the coronal plane to reveal neuroepithelial domains and cell migrations (*arrows*) that populate different olfactory cortical structures. *CoA* cortical amygdaloid nucleus, *LP* lateral pallium, *dLGE* dorsal lateral ganglionic eminence, *vLGE* ventral lateral ganglionic eminence, *MGE* medial ganglionic eminence, *OT* olfactory tubercle, *PC* piriform cortex, *RMTW* rostromedial telencephalic wall, *VP* ventral pallium

Lineage tracing using vital dyes, genetic approaches, or in utero electroporation in the mouse reveals an array of distinct VZ domains in the forebrain that contribute to the OC (Fig. 5; Table 1). These include the LP, VP, and dorsal and ventral segments of the LGE (dLGE and vLGE, respectively), which migrate together in the LCS, MGE, septum, RMTW, dorsal telencephalon, caudal telencephalon, and the diencephalon–telencephalon boundary (DTB). We will now discuss the different components of the OC formed as a result of migrations from these domains of origin.

**Table 1:**
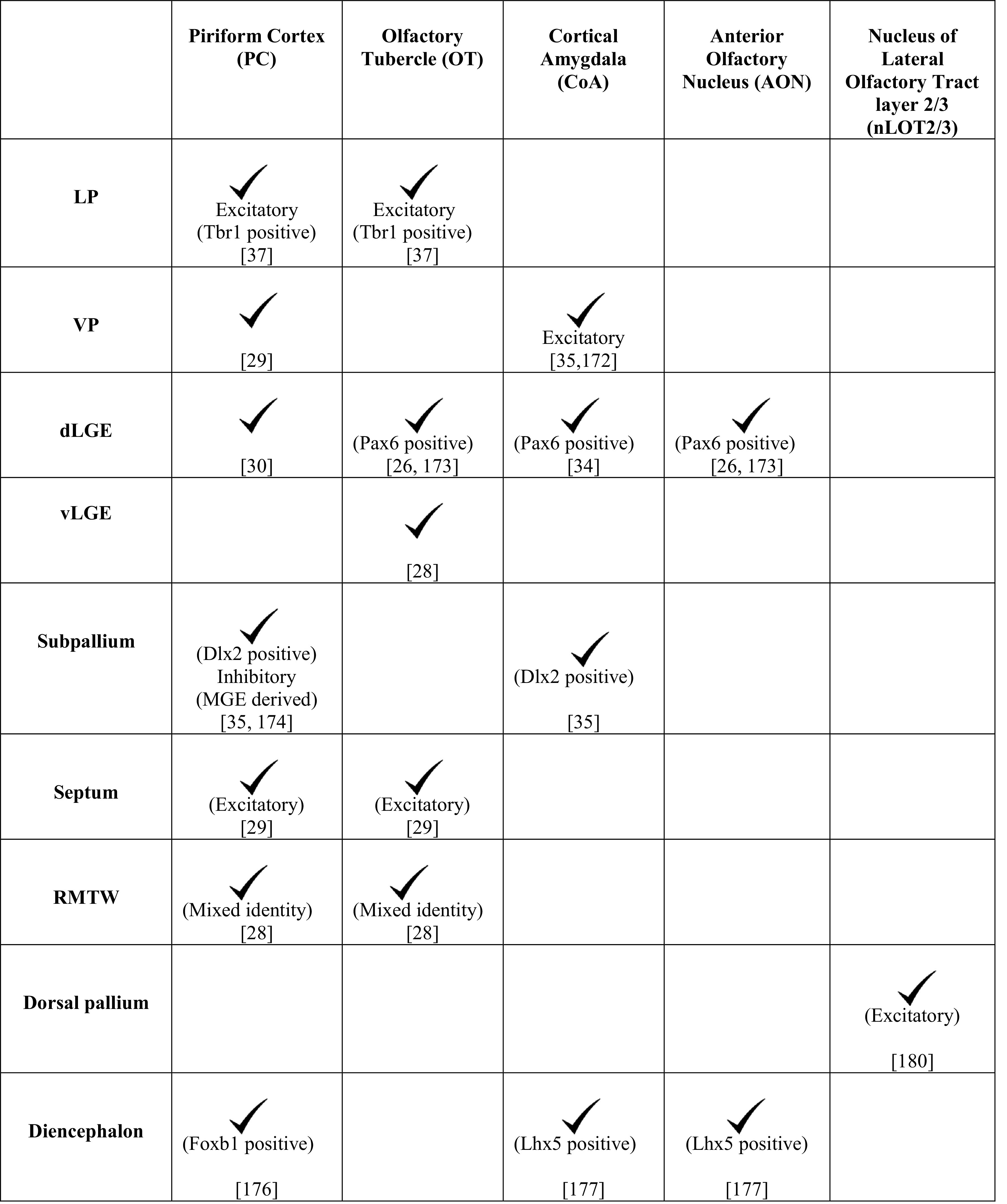
Domains of origin for the components of the OC

The different pallial and subpallial components of the LCS contribute excitatory and inhibitory neurons, respectively, to distinct structures of the OC (Table 1). A *Tbr1*-positive population arising from the LP contributes excitatory neurons to the OT and PC [37] whereas the VP produces excitatory projection neurons for the CoA [35, 172]. *Pax6*-positive interneurons from the dLGE are suggested to contribute to the AON, OT [26, 173], and the anterior CoA, which is reduced in the *Pax6* ^sey/sey^ mutant [34]. Cells migrating from the dLGE [30] and VP [29] also contribute to the PC, whereas neurons born from the vLGE contribute only to the OT (Fig. 5) [28]. The interneurons of the LCS display different modes of migration. A subset of the Dlx2-expressing population follows chain migration and proliferates en route to the CoA and PC [35], similar to the cells in the RMS. Interneurons migrating to the PC also originate from the MGE [174], a well-characterized source of cortical interneurons [175].

The PC and OT have a contribution from a rostral origin consisting of excitatory neurons arising from the septum [29] and neurons of mixed identity from a pallial domain immediately dorsal to it, called the RMTW (Fig. 5; Table 1) [28]. The PC also receives cells from the dorsal telencephalon [169] and a diencephalic population of the *Foxb1*-lineage [176]. An unusual population of cells exhibiting a tangential, surface migration arises caudally, possibly at the DTB, and migrates along the lateral aspect of the telencephalon to populate the rostral OC. This population expresses *Lhx5* like the DTB and displays similarities with Cajal–Retzius cells in its surface migration and *Reelin* expression [177].

#### Cell migrations to the nLOT

The nLOT is a trilaminar component of the olfactory amygdala and is bidirectionally connected to the OB and PC. It is implicated in non-pheromonal olfactory behaviors, especially feeding or ingestive behavior [178, 179]. Layers 2 and 3 of the nLOT (nLOT2/3) are the major output layers of this excitatory nucleus [179]. Whereas most of the amygdala develops either from the PSB or the subpallium, in utero electroporation of the caudal telencephalic neuroepithelium showed that the nLOT2/3 develops from the DP and therefore shares its origin and mechanisms of development with the neocortex [180]. Consistent with this interpretation, transcription factors required for proper development of the neocortex, such as Tbr1, Lhx2, and Pax6, are also required for the specification of the nLOT2/3 [34, 180, 181]. The neurons of the nLOT2/3 migrate along the caudal amygdaloid stream (CAS) and follow two modes of migration sequentially: a radial glia-independent phase that is parallel to the ventricular surface, followed by migration along the radial glia to their destination. Interestingly, this second phase requires Reelin and Cdk5 [180] similar to neurons of the neocortex [182, 183].

**Fig. 6:**
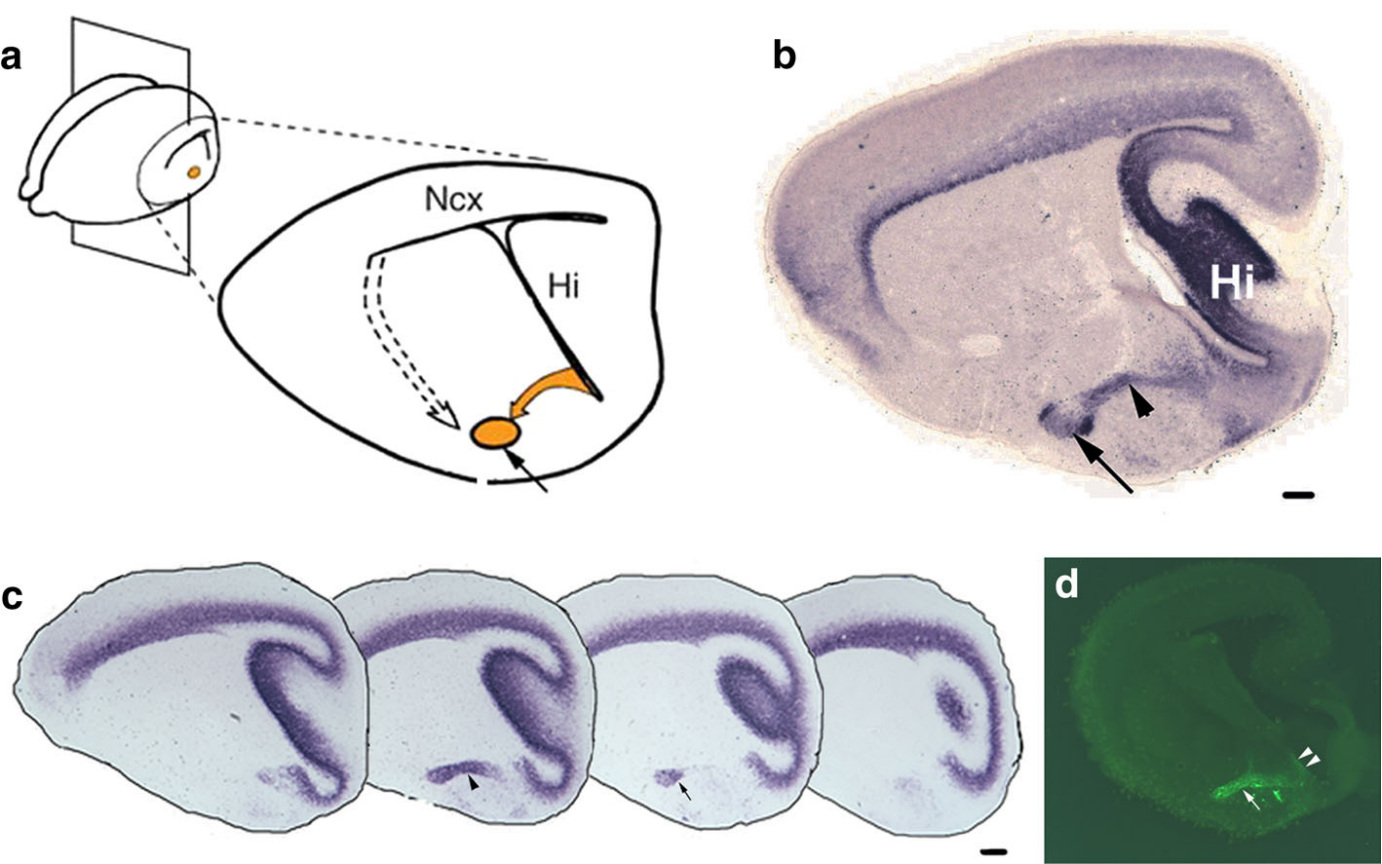
The caudal amygdaloid stream and migration to the nLOT2/3 [180]. **a** A sagittal section of an E17.5 mouse brain shows the caudal amygdaloid stream (CAS; *yellow arrow*) arising in the caudal telencephalic VZ and terminating in the globular nLOT2/3 (*yellow circle*, *black arrow*). *Dashed arrow* depicts the migration route from the VP to other amygdaloid nuclei. **b** Both the CAS (*arrowhead*) and the nLOT2/3 (*arrow*) are identified by *NeuroD* expression, **c** *NeuroD* expression in a lateral-to-medial series of sagittal sections at E15.5, **d** in utero electroporation of an EGFP-expressing construct in the caudal telencephalic neuroepithelium at E11.5, and examination of the brain at E15.5, reveals GFP-positive cells migrating along the CAS (*white arrow*). Note the residual GFP-positive neuroepithelium at the site of electroporation indicating the origin of the nLOT2/3 cells (*white arrowheads*). *Scale bars* are 200 μm. *nLOT* nucleus of lateral olfactory tract, *Hi* hippocampus, *Ncx* neocortex. All images in this figure are from [180], copyright 2007 Nature Publishing Group. Reprinted with permission

During development, the nLOT2 and 3 are indistinguishable [181], but on maturity, the nLOT3 appears as an ovoid structure surrounded by the crescent-like nLOT2 (Fig. 6). It would be useful to elucidate whether the origins of the nLOT2 and nLOT3 are indeed distinct from each other, since no fate-mapping study, either using genetic drivers or electroporation, distinguishes these two sub-nuclei. Layer 1 of the nLOT (nLOT1) is born at E10.5, a day earlier than the nLOT2/3 [180] and its origin and migration route are not well understood. The nLOT1 and nLOT2/3 express mutually exclusive markers and utilize distinct developmental mechanisms [33, 34, 179–181]. Therefore, the nLOT1 and nLOT2/3 may in fact be distinct nuclei that happen to assemble in close proximity gaining the collective name “the nucleus of the LOT.”

## The accessory olfactory system

Though the MOB has been traditionally thought to process common odors and the AOB to process pheromonal odors, there is increasing evidence of cross-talk between these two arms of the olfactory system at multiple stages, including the OB, amygdala and OC [184–188]. However, here we will treat the accessory system as a distinct entity, since this is more appropriate from a developmental perspective. Pheromonal odors are detected by vomeronasal sensory neurons (VSNs) in the vomeronasal organ (VNO). VSNs project via the olfactory nerve to the M/T cells, which are the projection neurons of the AOB. The M/T cells in turn project along the LOT together with the MOB axons. Targets of the AOB are nuclei of the vomeronasal amygdala, which in turn project to specific regions of the hypothalamus (Fig. 2) [189, 190]. We will describe cell migrations to each structure in the accessory olfactory circuit in the sections below.

### The accessory olfactory bulb

In rodents, the AOB is located on the dorsal aspect of the MOB and is the first recipient of vomeronasal innervation in the brain. VSNs project to the AOB in an ordered fashion such that the apical neurons project to the anterior AOB (aAOB) and basal neurons to the pAOB [191, 192]. The aAOB and pAOB also express a battery of mutually exclusive molecular markers and display an apparent functional segregation, such that the aAOB mediates mating behavior whereas the pAOB processes defensive/aggressive cues [16]. A developmental rationale for this functional dichotomy was provided by the discovery that the projection neurons of the aAOB and pAOB are produced in independent and widely separated domains of origin. Projection neurons of the aAOB originate from the local OB neuroepithelium, similar to those of the MOB. In contrast, projection neurons of the pAOB arise in a distant location at the DTB, migrating the entire rostrocaudal extent of the telencephalic surface to reach their destination [193].

The AOB was presumed to share developmental mechanisms with the MOB, in part due to the close juxtaposition and similar cellular composition of these two structures [194, 195]. However, the identification of disparate origins of aAOB and pAOB projection neurons implies that they may utilize distinct mechanisms for their development. Indeed, the aAOB requires mechanisms of specification that are similar to the MOB, whereas the pAOB depends on a different set of regulatory genes, e.g., *Tbr1* is required for the specification of the aAOB and MOB, but not the pAOB. The opposite is true for *Lhx5*, which is required for the specification of the pAOB, but not the aAOB or MOB [47, 193]. One unusual feature of pAOB neurons is that they perform tangential migration, a feature characteristic to interneurons that migrate over large distances [196] and not usually seen in other populations of projection neurons which migrate radially from the local VZ. Intriguingly, Cdk5, a molecule required for cell shape changes and necessary for radial glia-dependent migration [180, 183, 197] is also necessary for the pAOB neurons to migrate tangentially along the telencephalic surface, in the absence of which they remain accumulated at the caudal telencephalic pial surface [193].

The dichotomy between the domains of origin of the aAOB and pAOB is seen only for projection neurons. Interneurons of both the aAOB and pAOB are derived from the rostral LGE and from the anterior SVZ, similar to those populating the MOB [198, 199].

### The vomeronasal amygdala

Gene expression and fate-mapping studies have identified an assortment of nuclei of the amygdala and the extended amygdala [200, 201] to be part of the vomeronasal amygdala [189, 202–204]. These nuclei are all part of the vomeronasal circuit. The axons of AOB M/T cells project along the LOT to distinct nuclei of the vomeronasal amygdala. The MeA is the primary target of the AOB. Other targets include the posteromedial cortical nucleus (PMCo; Fig. 2) and components of the extended amygdala such as the bed nucleus of stria terminalis (BST) and the bed nucleus of the accessory olfactory tract (BAOT), all of which comprise the vomeronasal amygdala [190, 205].

#### Migrations to the MeA and PMCo

The MeA is anatomically divided into anterior, posterodorsal, and posteroventral divisions (MeAA, MeAPD, and MeAPV, respectively), which send projections to functionally distinct hypothalamic nuclei. The MeAPD projects to the reproductive hypothalamic nuclei and the MeAA and the MeAPV project mainly to the nuclei processing defense and aggression [189, 206, 207].

**Fig. 7:**
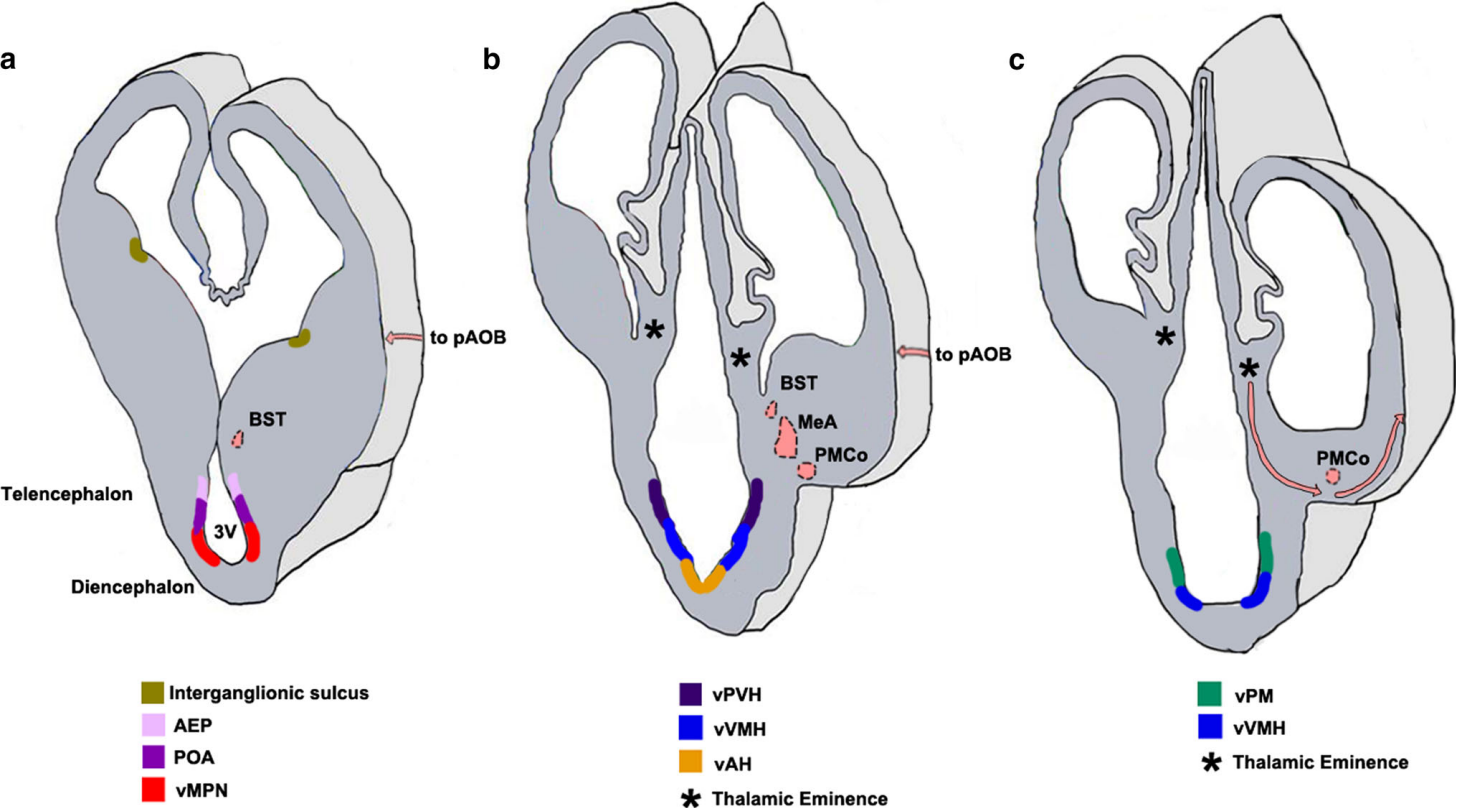
Neuroepithelial domains contributing to components of the vomeronasal system [36, 193, 203, 204, 212–214, 225, 226]. **a**–**c** Schematics of sections of the forebrain at three rostrocaudal levels in the coronal plane showing the different neuroepithelial domains that contribute to the hypothalamic nuclei of the VNS. Newborn neurons migrate radially from the designated neuroepithelial ventricular zones (vMPN, vAH, vVMH, vPM) to populate the MPN, AH, VMH, and PM, respectively. **a** The AEP and POA provide neurons to the BST, MeA, and PMCo. The interganglionic sulcus, between the LGE and MGE, generates interneurons destined for the AH/POA. **b** The vPVH produces neurons for the vomeronasal amygdala (MeA, PMCo and BST). **c** the TE (*asterisks*, **b**, **c**) generates M/T neurons destined for the pAOB, which migrate to the telencephalic surface at caudal levels (*pink arrows*). These neurons undertake a tangential migration along the telencephalic surface (*pink arrows*, **a**, **b**) to the rostrally located pAOB which is not seen in the schematic. The pAOB, MeA, PMCo, BST, and the hypothalamic nuclei are all generated from the VZ of the third ventricle and are all part of the VNS. *3V* third ventricle, *AEP* anterior entopeduncular area, *BST* bed nucleus of stria terminalis, *MeA* medial amygdaloid nucleus, *pAOB* accessory olfactory bulb, posterior division, *PMCo* posteromedial cortical nucleus, *POA* preoptic area, *TE* thalamic eminence, *vAH* ventricular zone for anterior hypothalamic nucleus, *vMPN* ventricular zone for medial preoptic nucleus, *vPM* ventricular zone for pre-mammillary nucleus, *vPVH* ventricular zone for paraventricular hypothalamic nucleus, *vVMH* ventricular zone for ventromedial hypothalamic nucleus

In utero electroporation, lineage tracing using vital dyes, and genetic mapping studies show that each of these subnuclei is populated by cells from multiple pallial and subpallial neuroepithelial domains (Table 2). The *Lhx9* expressing VP populates both the MeA and PMCo [33, 202]. The rostral portion of the VP contributes to the MeAA and a more caudal portion of the VP contributes to both the MeAPD and the MeAPV [29, 202, 204]. The MeAA receives neurons of the *Nkx2.1* lineage [211] from the subpallial anterior entopeduncular area (AEP) and preoptic area (POA). In contrast, the MeAPD is populated by cells of the *Nkx2.1* lineage from the AEP, and the MeAPV receives Dbx1-positive neurons of the *sonic hedgehog* (*Shh*) lineage from the POA (Fig. 7) [173, 202, 204, 209]. A population of glutamatergic neurons of MeA and PMCo is derived from the third ventricle. These cells express a diencephalic transcription factor, orthopedia (Otp), which is necessary for their migration across the DTB [203]. This migration takes place along radial glia which extend from the neuroepithelium underlying the paraventricular hypothalamic nucleus (PVH) to the MeA [36, 202–204].

**Table 2:**
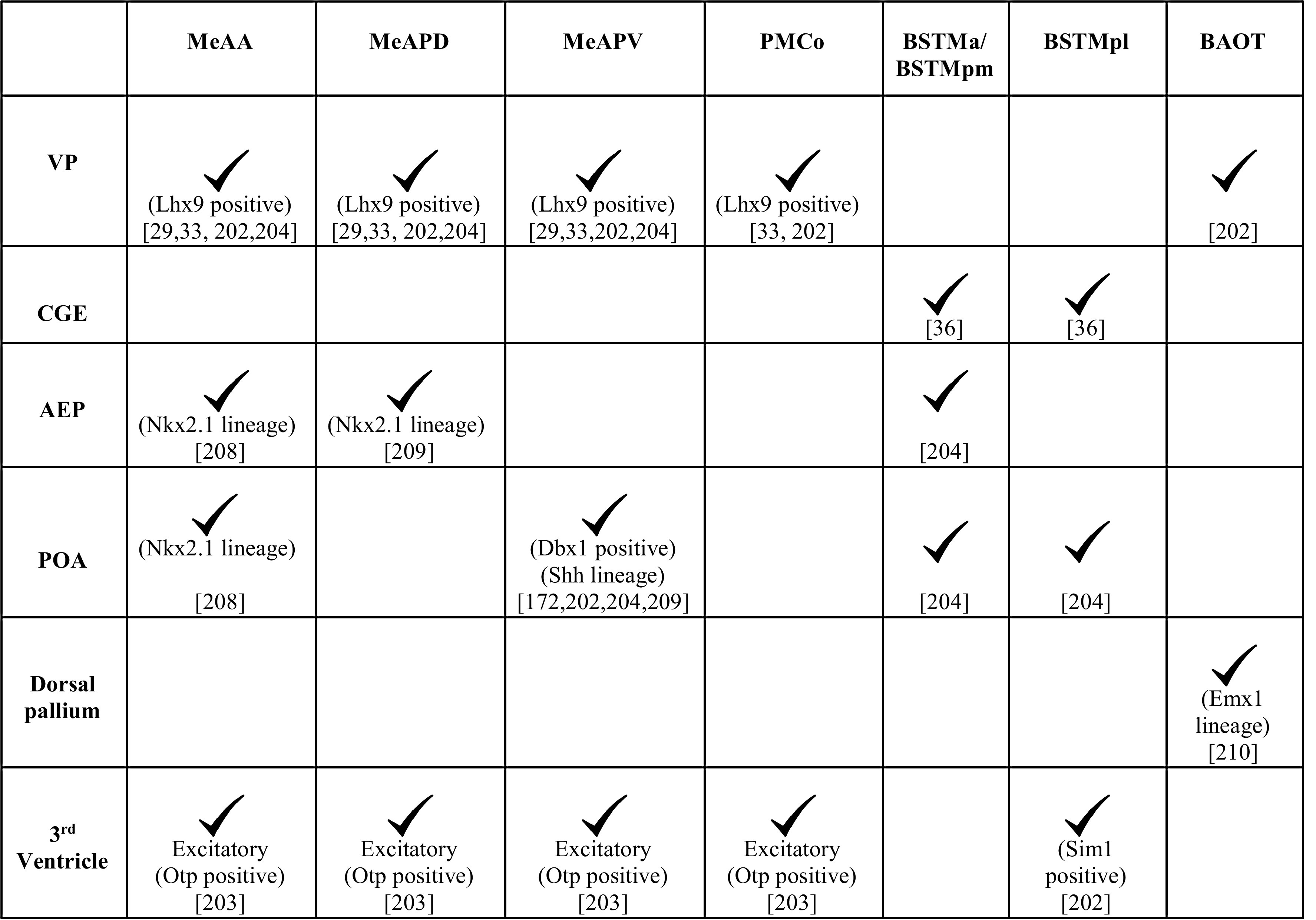
Domains of origin for the components of the vomeronasal amygdala

#### Migrations to the BST and BAOT

The BST is an important relay structure and a target of the MeA [187, 189]. It is divided into lateral (BSTL) and medial components (BSTM) which are involved in mediating autonomic and vomeronasal responses, respectively. The BST has heterogeneous origins that have been revealed by lineage tracing using vital dyes, in utero electroporation, and genetic mapping studies (Table 2) [36, 202–204].

Neurons of the BSTM are derived from the AEP, commissural POA, CGE and the neuroepithelium of the third ventricle underlying the PVH (Fig. 7) [36, 203, 204]. The third ventricle-derived population is part of the *Otp*-dependent excitatory neuron migration that also populates the MeA and the PMCo, described earlier [203]. The BSTM is parcellated functionally into anterior (BSTMa), posterolateral (BSTMpl), and posteromedial (BSTMpm) components. Studies based on gene expression patterns suggest that BSTMa and BSTMpm receive cells in large part from the AEP. The diencephalic population is thought to contribute largely to the BSTMpl based on the expression of *Pax6* and *Tbr1* in this subregion, and their coexpression with *Sim1* at the hypothalamic neuroepithelium [202].

BAOT, a lesser studied component of the vomeronasal amygdala, displays a distinct developmental profile from the other three components. Based on gene expression studies, cells for the BAOT appear to be derived from the VP [202] and the *Emx1*-lineage [210], indicating a pallial origin for this nucleus.

### Hypothalamic nuclei

The hypothalamus is a rostral diencephalic structure that receives input from a variety of systems including the olfactory system. In particular, the MeA projects densely to the medial preoptic nucleus (MPN), anterior hypothalamic nucleus (AH), ventromedial hypothalamic nucleus (VMH), and premammillary nucleus (PM). Of these, the MPN, ventrolateral VMH, and ventral PM process reproductive cues, whereas the AH, dorsomedial VMH, and dorsal PM process defensive/aggressive cues [187, 189, 211]. With the exception of the MPN, described below, the entire hypothalamus is largely born from the neuroepithelium of the third ventricle between E10 and E16 in mice (Fig. 7) [212, 213]. Newborn neurons migrate along radial glia to their respective positions in an outside-in pattern, such that the earliest born neurons migrate the farthest from VZ [214, 215].

#### Migrations to the hypothalamic nuclei

The MPN is a sexually dimorphic nucleus located in the POA and is considered to be more of a boundary structure at the DTB than a diencephalic region. In contrast to the migration pattern seen in the rest of the POA or other hypothalamic nuclei, the MPN develops using an inside-out pattern of radial migration similar to that seen in the dorsal telencephalon [214, 216]. The MPN also hosts neurons arising from an unusual migration from an unexpected source: GnRH neurons that control reproductive behavior, arising from the OP. These neurons undergo what may possibly be the longest tangential migration in the forebrain, arising at E10.5 in the OP. They undertake an axonophilic migration from E11.5 to E13.5, along a transient branch of the vomeronasal nerve from the VNO, penetrating the cribriform plate as part of the “migratory mass” described in an earlier section. They eventually settle in the MPN and lamina terminalis of the hypothalamus by E16.5 [108]. Not surprisingly, proper migration of GnRH neurons requires multiple guidance cues en route: adhesion molecules such as PSA-NCAM [217]; anosmin-1, an extracellular glycoprotein [218, 219]; semaphorin 4D–plexin B1 coupling [220]; ephrin receptor EphA5 [221]; and fibroblast growth factor 8 [222, 223]. The mechanisms of this complex migration have been reviewed elsewhere [224]. Once they settle in the MPN, they extend their axons into the median eminence thereby controlling the release of anterior pituitary hormones responsible for reproductive behavior.

The AH is located caudal to the POA with which it shares several developmental characteristics. The embryonic AH and the POA display a complementary expression of *Foxd1* and *Foxg1*, respectively. There are, however, some *Foxd1* expressing cells sprinkled in the AH, suggesting a possible migration of cells from the POA [225]. Though the closest ventricular domain to the AH and POA is the third ventricle, both structures receive radially migrating as well as tangentially migrating cells of telencephalic origin. Migrating cells are reported along the radial glia extending from telencephalic lateral ventricles adjacent to the septum and terminating at the pial surface of AH/POA [215]. Interneurons for the AH/POA migrate tangentially from the telencephalic inter-ganglionic sulcus between the LGE and the MGE, guided by nuclear receptors COUP-TF I and II [226]. The POA and the ventral midline of the AH both express *Shh* embryonically. The AH appears to be critically dependent on this factor, since the entire anterior hypothalamic area is severely reduced in the hypothalamus-specific knockout of *Shh* [227].

The VMH is a midline structure of the tuberal hypothalamic region and is derived from *Nkx2.1* expressing neuroepithelium [225] from E10 to E15 in mice [212]. Neurons that populate this region are from the *Shh*-lineage, arising from the neuroepithelium lining the third ventricle, and migrating along the radial glia to the mantle [228]. Though the VMH neurons all appear to arise from this single domain, the mature VMH contains molecularly distinct populations of cells in its different sub-regions. Neurons of the ventrolateral VMH express the estrogen receptor, ERα, and also the GABA_A_ receptor subunits. Neurons of the central and dorsomedial VMH express GABA_B_ receptor subunits. Not surprisingly, GABA plays an important role in regulating migration and positioning of the neurons, a function that is specific to VMH neurons and not those of adjacent hypothalamic nuclei [213, 229–231]. Central and dorsomedial VMH neurons also release brain-derived neurotrophic factor (BDNF) and selectively express an orphan nuclear receptor, steroidogenic factor SF-1/*Nr5a1* which is required for the positioning and coalescence of both SF-1 expressing and non-expressing VMH neurons [230, 232].

The PM is located in the mammillary region and is the most caudal of all the vomeronasal hypothalamic targets. Neurons of the PM are produced in the *Nkx2.1* positive neuroepithelium of the third ventricle. *Lef1*, a mediator and a target of Wnt signaling, is detected specifically in the PM neuroepithelium at the time of hypothalamic neurogenesis as well as in differentiated PM neurons later [225]. Wnt signaling may therefore play an important role in the formation of the PM. *Shh* is necessary for the differentiation of the PM, though its role for the AH and VMH development may be more prominent, since these latter nuclei are more severely affected in the absence of *Shh* [225, 227, 233, 234].

## Insights from olfactory system migrations: a developmental perspective

### Creative use of boundaries

A common developmental feature across all systems in which developmental patterning is examined is that cell lineage restriction boundaries or “compartment” boundaries play critical roles in defining the identities of adjacent regions. Boundaries prevent the intermixing of cells with other compartments, thereby spatially restricting cells destined to form particular structures and providing signaling cues to surrounding cell populations. When certain cell populations cross such boundaries, they add a layer of complexity to the system and deserve special attention. The olfactory system is rich in intriguing examples of this. Three boundaries in the forebrain have been well studied: the PSB in the telencephalon; the DTB at the telencephalic–diencephalic boundary; and the zona limitans intrathalamica (ZLI) in the diencephalon that demarcates the thalamus from the prethalamus. Each of these are known or proposed to be signaling centers that emanate cues to adjacent domains [26, 235–240].

The DTB has recently been proposed to be part of a “forebrain hem system” [27] and is witness to many migrations in each direction. Cells from the TE that cross the DTB and enter the telencephalon include cells destined for the pAOB, MeA, and OC. Lot cells that form a corridor in the lateral telencephalon to guide the axons of the LOT may also arise from the TE. Migrations in the opposite direction include cells arising from telencephalic domains that migrate across the DTB to the diencephalic AH and POA. The role of the boundary itself in these migrations is worth close examination in future studies.

The PSB is suggested to be a telencephalic signaling center [241, 242]. The domain abutting the PSB on the pallial side, the VP, expresses a number of morphogens and guidance cues such as the secreted form of Wnt antagonist, soluble Frizzled-related protein, *sfrp2* [236], chemokine SDF1 [243], and members of epidermal growth factor (EGF) family [241, 244]. Multiple migrations to components of olfactory system, the pAOB projection neurons [193], lot cells [153] (Ruiz-Reig et al., under revision, cerebral cortex), *Lhx5*-positive cells migrating to the rostral OC [177], and the axons of the LOT themselves follow a trajectory that traces the PSB on the lateral aspect of the telencephalon and may utilize cues secreted from this structure for their migration.

The olfactory system therefore illustrates that “boundaries” can play a wide range of roles in the development not necessarily limited to the canonical definition of segregating compartments.

### A hypothesis for programming connectivity at the domain of origin

Is connectivity between the individual components of a circuit encoded in their progenitors? Sokolowski and Corbin [195] proposed an elegant mechanism for the formation of complex circuits. They propose that if progenitors from a single domain expressing a specific set of transcription factors give rise to multiple structures of a circuit, the shared molecular code of the neurons may provide a mechanism to mediate the establishment of connectivity between those structures. There are some circuits in which such a mechanism could be examined. One example is the reproductive arm of the accessory olfactory system, in which several nuclei express *Lhx6* [189]. These include the MeAPD division of the MeA as well as its target, the BSTMpm division of the BST [187, 189], both of which arise from a common domain of origin, the AEP [202, 204], and connect to each other as part of a functional circuit.

Another example is the AOB–MeA circuit. In mouse and *Xenopus*, cells originating in the TE encounter the MeA in their migration route that ends in the AOB [193] and may contribute to it. The TE, migrating neurons, MeA, and AOB all express *Lhx5*. It may not be mere coincidence that the MeA is a major target of the AOB. Rather, their connectivity may be linked to a common domain of origin, the TE, which itself is known to control olfactory processing in amphibians [245].

Similarly, cell migrations from closely juxtaposed domains at the DTB populate many interconnected components of the VNS. The TE, MPN and POA are all located at the DTB in rodents (Fig. 7), in close proximity with the hypothalamic ventricle from which all of the hypothalamic targets of the VNS arise, indicating that much of the vomeronasal circuit originates from the diencephalic ventricle/DTB.

The olfactory system therefore offers an appropriately complex set of circuits to examine the hypothesis that the connectivity of a circuit may be linked to the domain of origin of its components. An exciting parallel has been demonstrated at a clonal level in the neocortex, wherein neurons born from the same progenitor preferentially connect to each other [246]. This process can have implications on function, e.g., sibling neurons in the primary visual cortex respond to similar visual stimuli for both orientation and direction [247]. Future studies could aim at finding downstream targets which direct connectivity between individual structures in the olfactory system.

## Insights from olfactory system migrations: a disease perspective

Understanding the nature and the mechanisms of cell migration in the olfactory system is critical for an insight into the etiology of disorders such as Kallmann syndrome, which is characterized by hypogonadotropic hypogonadism and anosmia. This disorder is caused by a failure of olfactory nerve formation, which results in a migration defect in GnRH neurons. There is concomitant aplasia of the OB itself, since pioneer axons from the olfactory nerve are known to stimulate OB evagination [30, 248, 249], causing defects in olfaction [218, 219, 250]. Mutations in *KAL1*, which encodes an NCAM anosmin-1, result in the X-linked form of the disease. Mutations in *KAL2*, which encodes the fibroblast growth factor receptor FGFR1, leads to the autosomal dominant form of Kallmann syndrome [219, 250–252].

Other developmental pleiotropic diseases such as the CHARGE syndrome, trisomy 13 or Patau syndrome, and trisomy 18 or Edward syndrome in which the OB is hypoplasic or aplasic also show migration defects in GnRH neurons [253], which leads to a decrease in the levels of circulating sex hormones causing hypogonadism in these patients. In contrast, when there is hyperplasia of the OB, it results in a different set of disorders such as fetal immobility/Pena–Shokeir syndrome. The enlarged OBs display lamination defects and the absence of glomeruli [254, 255]. In addition to developmental disorders, a number of neurodegenerative disorders are accompanied by deficits in proliferation or migration of neuroblasts from the SVZ to the OB. These deficits may be used for predicting the onset of disorders such as Alzheimer’s disease [256]. The hypothalamus is the seat of neuroendocrine control for the body and, therefore, disruptions of migration to the hypothalamic components of the circuit may underlie eating disorders such as anorexia nervosa, bulimia nervosa, neurohypophyseal diabetes insipidus, disorders of sexual behavior, and mood disorders [257, 258]. Some patients with eating disorders show inability to detect and/or identify odors [259], which may be due to abnormal proliferation or migration of the olfactory components of the system, underscoring the olfactory–hypothalamic relationship in development. Understanding cell migrations in the olfactory system can therefore provide insights into disease etiology and treatment.

OECs, which envelop the OSN axons, are being evaluated as potential donor cells for transplantation therapy in peripheral nerve and spinal cord injuries [260–262]. OECs derived from the adult rodent OB express myelin-associated proteins and have been shown to myelinate axons of co-cultured dorsal root ganglion cells in vitro [263, 264]. Furthermore, in vivo OEC transplants in rodents appear to promote spinal cord regeneration and recovery of behaviors affected due to spinal cord injuries [265–267]. One study in humans performed intraspinal grafting of autologous OECs and fibroblasts isolated from the olfactory mucosa in paralyzed patients with complete spinal cord injury. Both control and transplant recipients received intense neurorehabilitation, but only the latter displayed some recovery of neurological function [262]. Therefore, migratory cells of the olfactory system may have properties that are not utilized in normal life, but may be harnessed in translational paradigms for therapeutic applications.

## Insights from olfactory system migrations: an evolutionary perspective

The olfactory system is the most ancient sensory system and is evolutionarily conserved in terms of function, connectivity, and development across vertebrates [268, 269]. The conservation of origins for different structures within the olfactory system across disparate vertebrate classes is remarkable. This is particularly evident in OB development. OB M/T cells are pallial, and OB interneurons are subpallial in origin in rodents, chicks, *Xenopus*, and fish [26, 270–276]. Several vertebrate species including fish [277–279], reptiles [280, 281], rodents [77, 78], and non-human primates [282, 283] demonstrate postnatal and adult olfactory neurogenesis and migration of neuroblasts along the RMS. Adult humans also have a proliferative SVZ [284, 285] and there is evidence of migrating olfactory neuroblasts along the RMS to the OB [286].

Some differences in the nature of RMS migration are intriguing and may hint at how this phenomenon evolved: neuronal precursors migrate along the RMS from the telencephalic VZ to the OB in the adult zebrafish brain [279, 287], but this migration is along radial glia and not surrounded by glial tubes [279, 288, 289], in contrast to the rodent RMS [78].

In rodents, the AOB is a distinct substructure from the MOB. This distinction is not observed in fish except lungfish [290, 291], nor is it seen in some reptiles such as crocodiles and turtles, and birds [292]. While amphibians do have an AOB, it does not appear to be divided into the aAOB and pAOB [273, 276] except in the common Japanese toad, *Bufo japonicus* [293]. However, projection neurons of *Xenopus* AOB originate in the TE and migrate from the caudal end of the telencephalon rostrally to the AOB, similar to the migration of mouse pAOB projection neurons [193]. This leads to the tantalizing speculation that the amphibian AOB may correspond to the pAOB in rodents, and that the rodent aAOB may be an added-on specialization derived from the MOB, with which it shares its domain of origin and migratory mechanisms [193].

Multiple amygdaloid nuclei are implicated in olfactory and vomeronasal behaviors in rodents [16, 167]. The MeA in particular has been studied across several species of tetrapods and anurans. In anurans, the MeA is the only nucleus that receives input from the olfactory system [294–296]. Consistent with its subpallial identity, the developing MeA expresses *Nkx2.1* in *Xenopus* and rodents [208, 209, 268, 274, 276]. However, the MeA also receives migratory cells from disparate sources, making it a highly mixed structure. In rodents and *Xenopus*, pallial origin cells from the VP marked by *Lhx9* expression populate the MeA [268, 274, 297]. In addition, cells from the diencephalic VZ also migrate to the MeA in rodents [36], and this migration requires *Otp* [203]. A similar migration is suggested by *Otp* expression in *Rana perezi* and axolotl [298].

The apparent diencephalic-origin, *Otp* expression, subpallium-derived *Nkx2.1*/*Lhx6* expression, and the VP-derived *Lhx9* expression are also seen in the MeA of reptiles [298–300] and chicks [26, 298, 301, 302], suggesting that multiple origins for the MeA are conserved in different vertebrate species. These observations bring the MeA to the center stage for studies of behavior arising from circuitry that may also be similarly conserved.

## Concluding remarks

The olfactory system mediates a variety of social, motivational, and emotional behaviors including innate behaviors that are important for the survival of an organism and for the propagation of its species. This fundamental purpose may explain why the organization of the olfactory circuit is similar across most vertebrates. This is also consistent with the considerable degree of conservation of developmental origins and cell migrations to diverse structures within the system [193, 298, 299]. The olfactory system is composed of both nuclear and laminated structures. The mechanisms that shape the assembly of this ancient sensory system may have laid the foundation of developmental mechanisms for evolutionarily more recent structures such as the neocortex.

The olfactory system presents a combination of ancient origins, complex migrations leading to intricate circuitry, evolutionarily conserved circuit components and regulatory mechanisms, and fundamental behaviors critical for the maintenance of a species. These features make the olfactory system an attractive model for understanding both, the developmental mechanisms of circuit assembly in the forebrain and the possible evolution of these strategies in more recent structures such as the neocortex. Insight into the mechanisms underlying disorders arising from aberrant olfactory system development may also inform our understanding of disorders arising from defective neocortical development.

## Abbreviations

AEP: Anterior entopeduncular area
AH: Anterior hypothalamic nucleus
AOB: Accessory olfactory bulb
aAOB: Anterior division, accessory olfactory bulb
pAOB: Posterior division, accessory olfactory bulb
AON: Anterior olfactory nucleus
aSVZ: Anterior sub-ventricular zone
BAOT: Bed nucleus of accessory olfactory tract
BST: Bed nucleus of stria terminalis
BSTL: Bed nucleus of stria terminalis, lateral division
BSTM: Bed nucleus of stria terminalis, medial division
BSTMa: Bed nucleus of stria terminalis, medial division, anterior portion
BSTMpl: Bed nucleus of stria terminalis, medial division, posterolateral portion
BSTMpm: Bed nucleus of stria terminalis, medial division, posteromedial portion
CGE: Caudal ganglionic eminence
CoA: Cortical amygdaloid nucleus
DTB: Diencephalic–telencephalic boundary
DP: Dorsal pallium
EGF: Epidermal growth factor
FGF: Fibroblast growth factor
LCS: Lateral cortical stream
LGE: Lateral ganglionic eminence
dLGE: Dorsal lateral ganglionic eminence
vLGE: Ventral lateral ganglionic eminence
LOT: Lateral olfactory tract
LP: Lateral pallium
MCL: Mitral cell layer
MeA: Medial amygdaloid nucleus
MeAA: Medial amygdaloid nucleus, anterior division
MeAPD: Medial amygdaloid nucleus, posterodorsal division
MeAPV: Medial amygdaloid nucleus, posteroventral division
MGE: Medial ganglionic eminence
MOB: Main olfactory bulb
MP: Medial pallium
MPN: Medial preoptic nucleus
nLOT: Nucleus of lateral olfactory tract
OBLS: Olfactory bulb-like structure
OC: Olfactory cortex
OT: Olfactory tubercle
PC: Piriform cortex
PMCo: Posteromedial cortical nucleus
PM: Pre-mammillary nucleus
POA: Preoptic area
PSB: Pallial–subpallial boundary
PSA-NCAM: Polysialic acid–neural cell adhesion molecule
PVH: Para-ventricular hypothalamic nucleus
RMS: Rostral migratory stream
RMTW: Rostromedial telencephalic wall
*Shh*: Sonic hedgehog
TE: Thalamic eminence
VMH: Ventromedial hypothalamic nucleus
VNO: Vomeronasal organ
VNS: Vomeronasal system
VP: Ventral pallium
VZ: Ventricular zone
ZL: Zona limitans intrathalamica
